# Improvement of Tardive Dyskinesias with Olanzapine

**DOI:** 10.1155/2023/6688623

**Published:** 2023-10-23

**Authors:** S. Echater, B. Oneib

**Affiliations:** Psychiatry Department, Mohammed VI University Hospital of Oujda, Faculty of Medicine and Pharmacy, Mohammed 1st University, Oujda, Morocco

## Abstract

Tardive dyskinesia (TD) is characterized by abnormal and involuntary movements that generally occur after prolonged exposure to neuroleptic medications. In this article, we present the case of a 29-year-old man with schizophrenia who developed TD following treatment with haloperidol. Despite various attempts with benzodiazepines, amantadine, and anticholinergics, the dyskinesias persisted. However, after 2 years of treatment with olanzapine alone, a progressive improvement occurred, leading to the complete disappearance of the dyskinesias. We also provide a brief review of reported cases of antipsychotic-induced TD that has improved with olanzapine.

## 1. Introduction

Tardive dyskinesia (TD) is an abnormal and involuntary movement that generally occurs after prolonged exposure to neuroleptic medications (duration of treatment equal to or greater than 3 months or 1 month for people over 60 years) or following discontinuation of treatment. These movements persist for at least 4 weeks and primarily affect the orofacial region, trunk, and/or limbs [1]. TD can present in various forms, such as choreiform movements (rapid, jerky, nonrepetitive), athetoid movements (sinuous, slow, continuous), or rhythmic movements (stereotypies) [2].

Studies have shown that atypical antipsychotics present a significantly reduced risk of TD compared to first-generation antipsychotics [3–5]. The incidence rates of TD under antipsychotic treatment vary significantly across studies. The prevalence of TD ranges between 24% and 32% with first-generation antipsychotics and around 13% with atypical antipsychotics [1]. Evidence suggests that the use of atypical antipsychotics may also alleviate symptoms of preexisting TD [6–8]. Initial research on clozapine has shown its effectiveness in reducing TD induced by first-generation antipsychotics [9, 10]. Subsequent studies on other atypical antipsychotics have also reported beneficial effects of these medications on TD [11–14]. Furthermore, there are several reports of TD associated with atypical antipsychotics that have improved after switching to another atypical antipsychotic [15].

Olanzapine, a chemically similar atypical antipsychotic to clozapine, is known to cause fewer extrapyramidal side effects and TD than haloperidol [16]. Moreover, several articles have reported marked improvement in some patients with TD after treatment with olanzapine [16–19].

We present a case of a patient who showed significant improvement in TD after 4 years of olanzapine treatment. We also provide a brief review of reported cases of antipsychotic-induced TD that improved after switching to olanzapine. This study aims to determine whether olanzapine can prove effective in treating TD.

## 2. Clinical Case

A 29-year-old man, single and without notable medical history, has been under psychiatric care since 2013 for schizophrenia. His first acute psychotic episode was characterized by a sudden onset of motor instability, agitation, insomnia, as well as persecutory, mystical, and referential delusions, along with auditory hallucinations. He was treated with a daily dose of 12 mg of haloperidol and 300 mg of chlorpromazine. During his first exposure to neuroleptics, he developed an acute dystonic reaction, which was successfully treated with 5 mg of trihexyphenidyl per day. After approximately 20 days of hospitalization, the patient experienced significant improvement and returned to his previous state of health. He was discharged from the hospital and began outpatient follow-up. Unfortunately, after about 7 months of treatment, the patient decided to discontinue his medication on his own and stopped attending follow-up appointments.

In 2014, 5 months after discontinuing his treatment, the patient experienced his second psychotic episode, leading to a second 1-month hospitalization, followed by subsequent improvement. However, after his discharge, he did not consistently adhere to his outpatient follow-up or treatment regimen. Nevertheless, the patient remained stable until 2016 when he began displaying early signs of a relapse, including insomnia, irritability, and persecutory thoughts. Additionally, he initiated tobacco and cannabis use.

Given this situation and due to the patient's poor treatment adherence, he was switched to haloperidol decanoate, a long-acting form of haloperidol, at a monthly dose of 150 mg. The option of using an atypical long-acting antipsychotic was not feasible due to its high cost and the patient's average socioeconomic status. The patient responded positively to this new treatment approach, experiencing a significant reduction in his psychotic symptoms. He continued to receive monthly injections of the long-acting treatment and was followed-up on an outpatient basis every 3 months.

One year after starting haloperidol decanoate, he gradually developed TD, characterized by brief, rapid, unpredictable, and variable-amplitude choreoathetoid movements. Clinical examination revealed facial grimaces and a combination of purposeless and repetitive movements affecting the upper and lower limbs, as well as the trunk. These manifestations prompted the patient's hospitalization in our psychiatric department to change his treatment to olanzapine. Psychiatrically, the patient was stable and did not exhibit overt delusions or perceptual disturbances. However, he displayed mild signs of depression related to his disabling motor symptoms. We initiated olanzapine at an initial dose of 10 mg/day, gradually increased to 20 mg/day.

However, despite this change in treatment, the dyskinesias persisted. The patient was then referred to a neurology specialist who conducted investigations such as a brain MRI, autoimmune screening, genetic testing, and thyroid function evaluation to rule out other causes of abnormal movements. The neurologist decided to initiate a treatment regimen for the patient, including 3 mg/day of alprazolam, 300 mg/day of amantadine, and up to 30 mg/day of trihexyphenidyl. Despite a slight improvement in the dyskinesias, the social and professional impact was significant. Therefore, the neurologist recommended tetrabenazine as a treatment option for the dyskinesias. Unfortunately, due to its unavailability in Morocco, the patient was unable to obtain this medication abroad. As a result, the patient maintained treatment with 20 mg/day of olanzapine and 300 mg/day of amantadine, with intermittent use of benzodiazepines. Trihexyphenidyl treatment was discontinued after approximately 1 month of prescription. The patient continued his follow-up in psychiatric and neurological consultations. On the psychiatric front, he experienced a schizophrenic relapse in 2018, characterized by delusional ideas and auditory hallucinations, following a reduction in his olanzapine dose to 15 mg/day by his treating physician. This relapse was managed on an outpatient basis, with treatment adjustments, including an increase in the olanzapine dose to 20 mg/day and the addition of a sedative neuroleptic, chlorpromazine, at a dose of 150 mg/day. Chlorpromazine was discontinued after improvement, which occurred after 2 months.

In February 2019, after nearly 2 years of treatment that included olanzapine at a dosage of 20 mg/day, amantadine at a dosage of 300 mg/day, and intermittent use of benzodiazepines characterized by periods of gradual discontinuation, resumption, new discontinuations, and intermittent use throughout the 2-year period, the patient did not observe significant improvement in his dyskinesias. Faced with this frustrating situation, he decided to discontinue all other medications and continue only with olanzapine at a daily dose of 20 mg. Two years later, in March 2021, the patient spontaneously and gradually started decreasing his dyskinesias, without any modifications in treatment or additional medical intervention. Over the following 6 months, the patient's dyskinesias completely disappeared.

On the psychiatric front, since the patient had been a good adherent to his olanzapine treatment at a dose of 20 mg/day, he remained stable and did not experience a relapse during these 2 years.

## 3. Discussion

In the present article, we describe the case of a patient who developed TD induced by first-generation antipsychotics but experienced almost complete improvement 2 years after treatment with olanzapine alone.

Spontaneous remission of TD remains a rare phenomenon that is poorly understood and insufficiently documented in the literature. In our case, it is possible to consider that the improvement in TD could have been spontaneous, independent of olanzapine treatment, especially since this improvement occurred 4 years after starting olanzapine treatment. However, it can also be considered that treatment with olanzapine contributed to this improvement. Previous studies have reported similar improvements with the use of olanzapine, such as the one conducted by Kinon et al. [13], a prospective double-blind clinical trial that demonstrated approximately 70% of patients treated with olanzapine at a dose of 5–20 mg/day, showed a significant reduction in TD symptoms after 8 months of treatment. Similarly, a study by Brar et al. [7] reported a statistically significant reduction in the prevalence and severity of dyskinetic symptoms in a group of 63 patients with schizophrenia, schizoaffective disorder, or bipolar disorder who were treated with olanzapine. In a publication by Lykouras et al. [17], two cases of patients with schizophrenia were described, showing complete remission of TD after 8 and 18 months of treatment with olanzapine, respectively. Other case reports have also described the improvement of TD following treatment with olanzapine (Table 1).

Most reported cases of dyskinesia remission occur within the first 5 weeks following olanzapine treatment, with the longest delay being 1.5 years (Table 1). However, our case stands out due to notable peculiarities. First, unlike other reported cases, our patient did not show improvement after discontinuing the implicated neuroleptic and initiating monotherapy with olanzapine. This observation highlights a deviation from the traditional patterns of remission observed in other cases.

Furthermore, it is worth noting that in most previously reported cases, rapid improvement following the switch to olanzapine monotherapy occurred within an average of 5 weeks, with the longest delay being 1.5 years. An isolated exception has been documented in the literature where a case showed improvement after combining olanzapine with tetrabenazine and tiapride [19].

In our case, however, the dynamics differ significantly. After switching to olanzapine, our patient did not experience improvement, and this lack of response persisted even during 2 years of concurrent treatment with amantadine and benzodiazepines. It was only after discontinuing both medications and continuing with olanzapine monotherapy for an additional 2 years that we observed substantial improvement in his symptoms

It is important to note that the patient voluntarily discontinued taking amantadine 2 years before observing an improvement in his dyskinesias, suggesting that this medication did not have a determining role in the symptom remission.

Olanzapine and clozapine have a moderate affinity for 5-HT3 receptors. Therefore, it is possible that the antagonistic effect of olanzapine on these receptors contributed to the improvement of the dyskinetic disorder [17]. Studies have indeed demonstrated that the administration of ondansetron, a 5-HT3 receptor antagonist, improves dyskinesias [27].

Furthermore, it should be noted that some studies have reported the development of TD in relation to the use of olanzapine and clozapine [28–33].

It is also possible to consider that the improvement in TD may have occurred spontaneously. However, in order to verify the effect of olanzapine on the improvement of TD, we could have discontinued olanzapine. Nevertheless, such a decision would have been unethical as it could have led to a relapse of schizophrenia.

Fernandez et al. [34] reported spontaneous resolution of TD in 33 out of 53 patients who were followed for 14 years. However, due to the limitations of the study, it is not possible to definitively conclude whether clozapine or other factors may have influenced the improvement of TD. The lack of information on disease duration, retrospective medication data, and the limited number of patients constitute the limitations of this study [34].

To our knowledge, there is currently no relevant large-scale clinical study specifically addresses the use of olanzapine in the treatment of TD. Although a few case reports have been published on this subject, further comprehensive studies are still needed to rigorously evaluate the effectiveness of olanzapine in managing TD.

## 4. Conclusion

The favorable response of TD to olanzapine in our patient suggests that olanzapine could be a useful alternative medication to clozapine. However, this question requires further in-depth investigations to confirm this hypothesis. Thus, we recommend conducting larger-scale randomized controlled trials aimed at specifically assessing the efficacy of olanzapine in treating TD. Additionally, prospective studies on a larger scale could be undertaken to provide a more comprehensive evaluation of olanzapine's effects on these motor disorders. Furthermore, it would be essential to conduct thorough research into the underlying mechanisms through which olanzapine exerts its beneficial effects on TD.

## Figures and Tables

**Table 1:** Cases of tardive dyskinesia (TD) induced by antipsychotics that were improved by olanzapine.

Author	Number of patients		Antipsychotic that induced TD	Olanzapine dose (mg/day)	Comedication	Time to TD improvement
Almeida [20]	One		Haloperidol	10		5 weeks
O'Brien and Barber [21]	One		Trifluopérazine, sulpiride, and rispéridone	5		6 months
Raja et al. [22]	Three	Case 1, Case 2, and Case 3	Haloperidol decanoate, haloperidol decanoate, and haloperidol	20, 10, and 15		2, 5, and 2 weeks
Haberfellner [23]	One		Flupenthixol decanoate	15		4 months
Esel et al. [16]	One		Zuclopenthixol, haloperidol, and pimozide	10		2 months
Agarwal and Kumar [24]	One		Risperidone	5		2 weeks
İpekçi and Birsöz [25]	One		Risperidone	10		2 weeks
Lykouras et al. [17]	Two	Case 1 and Case 2	Trifluopérazine and biperiden and haloperidol	17.5 and 10		8 months and one and half year
Kučerová [26]	Two	Case 1 and Case 2	Conventional antipsychotics and thioridazine and risperidone	10 and 15		12 days and 2 months
Sacchetti and Valsecchi [18]	One		Haloperidol	20		4 weeks
Koch et al. [19]	One		Halopéridol	NA	Tétrabénazine and tiapride	2 weeks

NA, not available.

## Data Availability

No data are available for this study.

## Additional Points

*Limitations*. The study is based on a single clinical case and has a retrospective design, which limits the generalizability of the results to a larger population. Confounding factors and uncertainty about the cause of improvement: Uncertainty exists regarding whether the observed improvement in tardive dyskinesia is attributable to olanzapine, spontaneous improvement, or previous treatments.
